# *Listeria monocytogenes* adenosine auxotrophs are impaired for intracellular and extracellular growth but retain potent immunogenicity

**DOI:** 10.1128/iai.00343-25

**Published:** 2025-09-05

**Authors:** Ying Feng, Mariya Lobanovska, Jenna Vickery, Jesse Garcia Castillo, Leslie Güereca, Shannon K. Chang, Michel DuPage, Daniel A. Portnoy

**Affiliations:** 1 Department of Molecular and Cell Biology, University of California Berkeley 1438, Berkeley, California, USA; 2 Department of Plant and Microbial Biology, University of California Berkeley 1438, Berkeley, California, USA; University of Illinois Chicago, Chicago, Illinois, USA

**Keywords:** pathogenesis, vaccines, *Listeria*, immunity, purines

## Abstract

*Listeria monocytogenes* is a facultative intracellular pathogen that has garnered attention as a potential cancer therapeutic due to its ability to induce robust cell-mediated immunity. To ensure safe clinical administration, deletion of certain genes, such as *actA*, has been used to attenuate *L. monocytogenes*-based vaccine strains while preserving immunogenicity. Here we explored the potential inclusion of a *purA* gene deletion to enhance the development of *L. monocytogenes*-based immunotherapy. The *purA* gene encodes adenylosuccinate synthetase, which catalyzes the conversion of inosine monophosphate to adenosine monophosphate (AMP), a critical step in the *de novo* biosynthesis of purines. Since nucleotide biosynthesis is critical for the survival and pathogenesis of many bacterial pathogens, we examined the requirements of *L. monocytogenes* AMP synthesis in tissue culture and animal infection models. The *purA* mutants were able to escape from phagosomes of bone marrow-derived macrophages but were highly defective for subsequent growth in the host cell cytosol. In contrast to wild-type bacteria, the mutants did not grow in human serum or sheep blood. In intravenously infected mice, *purA* mutants were highly attenuated, similar to *actA* mutants, but displayed distinct growth kinetics during the course of infection. Remarkably, the *purA* mutants exhibited different localization patterns across splenic immune cells and elicited a more potent CD8^+^ T-cell response compared to *actA* mutants. These results underscore the essentiality of AMP biosynthesis for *L. monocytogenes* pathogenesis and provide new avenues for developing safe *L. monocytogenes*-based vaccines and therapeutics.

## INTRODUCTION

*Listeria monocytogenes* is a rapidly growing, Gram-positive, facultative intracellular bacterial pathogen of humans and animals (1, 2) that is genetically tractable and has been extensively characterized in mouse models of infection and immunity (3). This pathogen enters host cells, including professional antigen-presenting cells, escapes from phagosomes, grows in the host cytosol without causing extensive host cell death, and secretes antigens and metabolites that induce robust host cell-mediated immunity (4). These features make *L. monocytogenes* an attractive platform for the development of live vector-based cancer immunotherapy (5). Attenuated strains, including *L. monocytogenes* with limited cell-to-cell spread (Δ*actA*), have been administered to more than 1,800 cancer patients (5, 6). However, the immunogenicity of *L. monocytogenes* cancer vaccines in clinical trials was not as robust as that observed in preclinical studies (6). An additional concern is that in rare cases, live extracellular bacteria were found in patients’ blood or growing on implants (7). As a result, the attenuated vaccine strains occasionally caused severe adverse events and consequently put clinical trials on hold (5, 8).

As a facultative intracellular pathogen, *L. monocytogenes* can thrive in extracellular environments during systemic infection (9, 10), causing a significant risk for the safe administration of this bacterium in humans. Extracellular niches that support the extracellular growth of *L. monocytogenes* include but are not limited to intestines, blood, and gallbladders (11). An engineered strain lacking the ability to grow in these extracellular niches would significantly improve the safety profile of *L. monocytogenes*-based therapies. We explored nutrients that were limited in extracellular environments and found that the average concentration of purine bases and nucleosides in plasma and other extracellular fluids is low, generally in the range of 0.4–6.0 µM (12). More importantly, *de novo* nucleotide biosynthesis was identified as the single most critical metabolic function required for bacterial growth in human blood (13). This inherent importance of purine biosynthesis makes it a promising target for the development of therapeutic approaches against bacterial infections (14) and for designing live, attenuated *L*. monocytogenes-based therapeutics (15–17).

**Fig 1:**
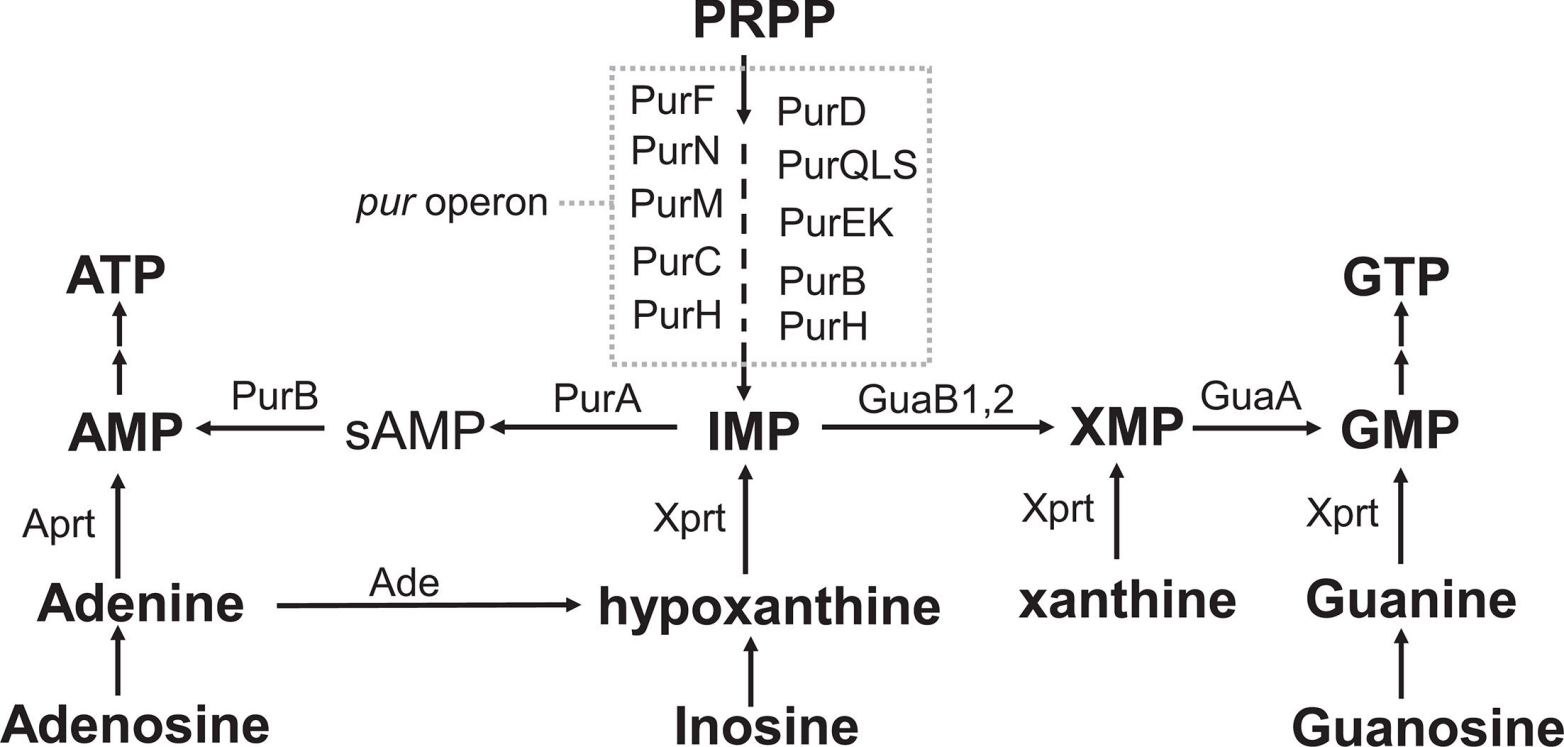
*L. monocytogenes* purine biosynthesis and salvage pathways. The pathways involve the *de novo* synthesis of inosine monophosphate (IMP) from phosphoribosyl pyrophosphate (PRPP) by the *pur* operon encoded enzymes and the subsequent synthesis of adenosine monophosphate (AMP) and guanosine monophosphate (GMP). Adenylosuccinate synthetase (PurA) catalyzes the conversion of IMP to adenylosuccinate sAMP, the intermediate product of AMP. Nucleotides can be made by the salvage of nucleosides and nucleobases through phosphoribosyltransferases, APRT, and XPRT (23) .

Purines participate in many cellular functions, including DNA/RNA biosynthesis, energy storage, and signal transduction. Given these fundamental roles, purine biosynthesis and acquisition are essential for all living organisms, including bacterial pathogens (18). Many bacteria synthesize nucleotides *de novo* but also import exogenous nucleobases and nucleosides. The *de novo* purine synthetic pathway involves the construction of a purine ring, resulting in the formation of inosine monophosphate (IMP), which can be further converted to adenosine monophosphate (AMP) and guanosine monophosphate through distinct branches of the biosynthetic pathway (Fig. 1). Notably, the conversion from IMP to AMP appears to be critical during *L. monocytogenes* infections. Genome-wide transposon insertion screens have identified the *purA* and *purB* genes, encoding enzymes responsible for the conversion of IMP to AMP (Fig. 1), as essential for the intracellular proliferation of *L. monocytogenes* in human epithelial Caco-2 cells (19) and murine J774 macrophage-like cells (20). Additionally, *purA* mutants of serotype 4b *L. monocytogenes* were severely impaired in their ability to colonize the gastrointestinal tract and cause systemic infections (21, 22). These findings underscore the importance of AMP synthesis for *L. monocytogenes* growth and virulence. However, we still lack detailed information on the role of AMP synthesis during *L. monocytogenes* pathogenesis and immune response.

In this study, we generated an *L. monocytogenes* adenosine auxotrophic mutant (Δ*purA*) to determine the role of *L. monocytogenes* AMP synthesis during infection and immunity. Although *purA* mutants were able to escape phagosomes, they failed to proliferate within the cytosol of murine bone marrow-derived macrophages (BMMs) *in vitro*. Additionally, the *purA* mutants did not grow in human serum or sheep blood and were severely attenuated in a mouse infection model. However, despite their avirulent phenotype, Δ*purA* mutants elicited a CD8^+^ T-cell response and conferred immune protection against wild-type (WT) *L. monocytogenes* in mice.

## RESULTS

### *L. monocytogenes* Δ*purA* mutants are auxotrophic for adenine and adenosine

The synthesis of AMP from IMP was proposed to be necessary for *L. monocytogenes* infection based on genome-wide screen in tissue culture models of infection (19, 20). However, our previous work showed that *L. monocytogenes* Δ*purEK* mutants, deficient in IMP synthesis (Fig. 1), behaved like WT in tissue culture models and were only moderately attenuated during *in vivo* infection (24). These findings led us to question the importance of purine biosynthesis in *L. monocytogenes* pathogenesis and prompted us to investigate the requirements of *L. monocytogenes* AMP synthesis during infection and immunity. IMP is converted to AMP by two sequential enzymatic reactions catalyzed by adenylosuccinate synthetase (PurA) and adenylosuccinate lyase (PurB) (Fig. 1). Since PurB is also involved in IMP *de novo* synthesis, we focused our analysis on characterizing *purA* mutants.

We generated an in-frame deletion mutation in *purA* (Δ*purA*) and constructed a complemented strain in which *purA* was transcribed using its native promoter (Δ*purA + purA*) cloned into a stable integration vector. When cultured in chemically defined media (25), the Δ*purA* mutants failed to grow unless supplemented with adenine or adenosine but not hypoxanthine, the nucleobase of IMP (Fig. S1). The minimal concentration of exogenous adenine/adenosine required to support optimal growth of the Δ*purA* mutants was between 100 and 500 µM (Fig. S1B and C), a concentration that does not normally exist in extracellular environments (12). In contrast, the purine auxotroph Δ*purEK* grew when supplemented with either hypoxanthine or adenine/adenosine (Fig. S1A). The growth of the Δ*purA* mutants in the absence of purines was restored in complemented strains (Fig. S1A).

### AMP synthesis is required for *L. monocytogenes* growth in bone marrow-derived macrophages

**Fig 2:**
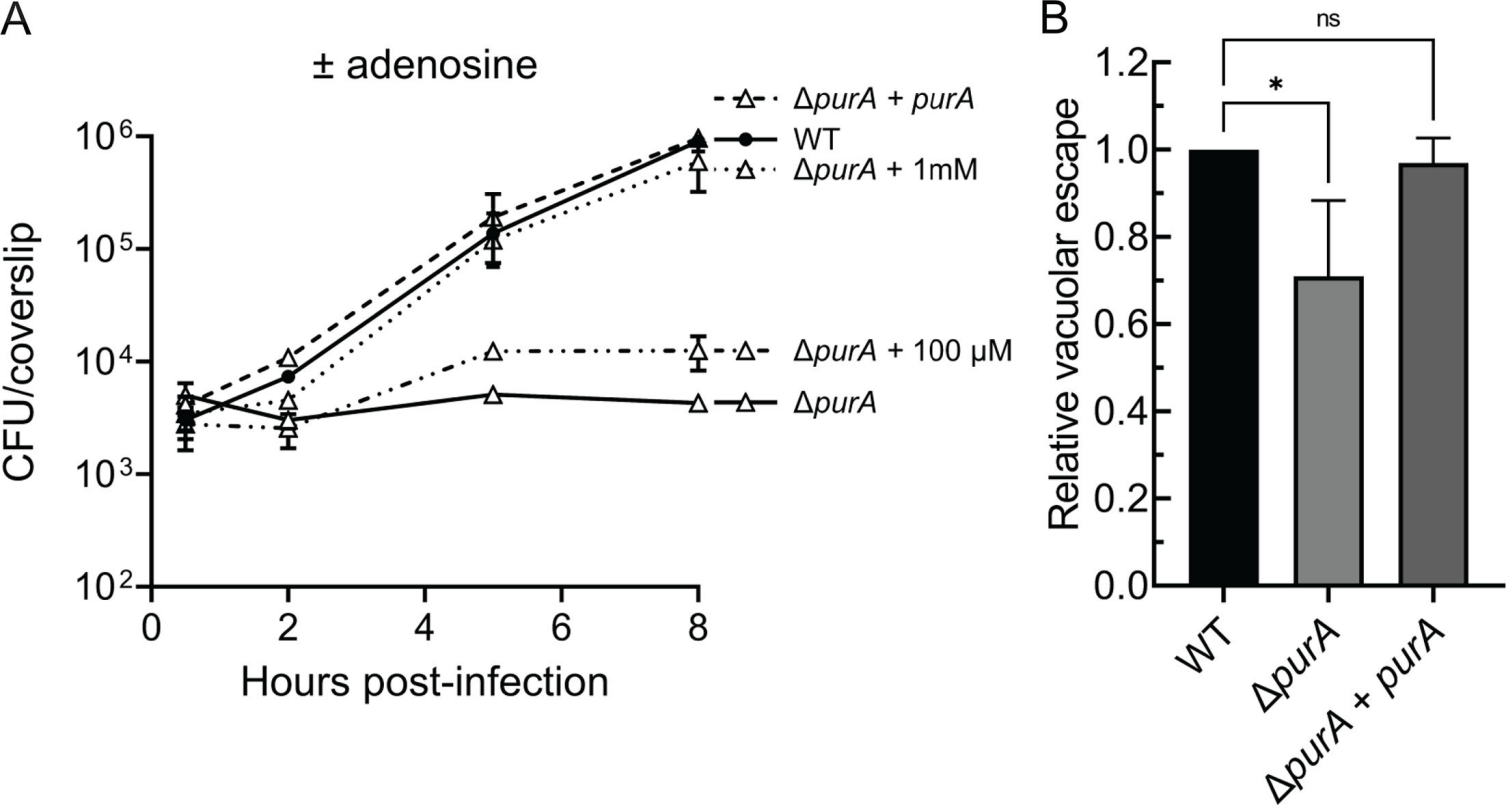
*L. monocytogenes* adenosine auxotrophs do not grow in BMMs. (**A**) Intracellular growth curve in BMMs. BMMs were infected at a multiplicity of infection of 0.25 for 30 min. Extracellular growth of bacteria was prevented by adding 50 µg/mL gentamicin at 1 hour post-infection. Adenosine was added to the BMM medium at 1 hour post-infection in the indicated groups. Growth was enumerated by plating the CFUs at 0.5, 2.0, 5.0, and 8.0 hours post-infection. (**B**) Relative phagosomal escape was measured by calculating the percentage of p62^+^ bacteria of the total intracellular bacteria for each strain and was normalized to that of WT. BMMs were infected with the indicated strains for 30 min. Data are mean ± SD. Three independent experiments were combined with more than 100 bacteria analyzed for each strain per experiment. One-way analysis of variance with multiple comparisons to WT was performed. ns, not significant. *P < 0.05.

During *L. monocytogenes* systemic infection, most bacteria are rapidly internalized and killed by resident phagocytic cells, but some of the bacteria escape from phagocytic vacuoles and replicate in the cytosol of infected cells, which is critical for *L. monocytogenes* pathogenesis as well as the establishment of host adaptive immunity to *L. monocytogenes* (3). Therefore, we determined the requirements of *purA* during infection of murine BMMs, a well-established phagocytic cell model for *L. monocytogenes* infection. We found that mutants lacking *purA* failed to grow in BMMs, while the WT and complemented strains had a more than 100-fold increase in growth during an 8 hour infection (Fig. 2A). The growth of Δ*purA* mutants was marginally improved when supplemented with 100 µM adenosine in the tissue culture media at 1 hour post-infection and was fully restored when an excess amount of adenosine (1 mM) was added (Fig. 2A).

We next asked if the lack of growth observed during Δ*purA* growth in BMMs was due to the inability of the mutants to escape from phagosomes. Immunofluorescent microscopy was used to distinguish phagosomal and cytosolic *L. monocytogenes* by colocalizing bacteria with a host cytosolic autophagy adaptor protein, p62 (26, 27). Once *L. monocytogenes* enters the host cell cytosol, it becomes coated with p62 when actin polymerization on bacteria surface is inhibited by cytochalasin D (28, 29). The Δ*purA* mutants were only partially impaired in phagosomal escape compared to the WT and the complemented strains, with approximately 70% phagosomal escape relative to WT (Fig. 2B). These results indicated that Δ*purA* mutants escaped from phagosomes but lacked sufficient adenine/adenosine to support their growth in the cytosol of BMMs.

We also explored the requirement of *purA* in an immortalized non-phagocytic L2 fibroblast infection model. In contrast to the findings in BMMs, the Δ*purA* mutants did not exhibit defects in plaque formation (Fig. S2), indicating that an adequate amount of adenine/adenosine was present in L2 cells that supported the replication of Δ*purA*.

### AMP synthesis is required for *L. monocytogenes* growth in blood and serum

**Fig 3:**
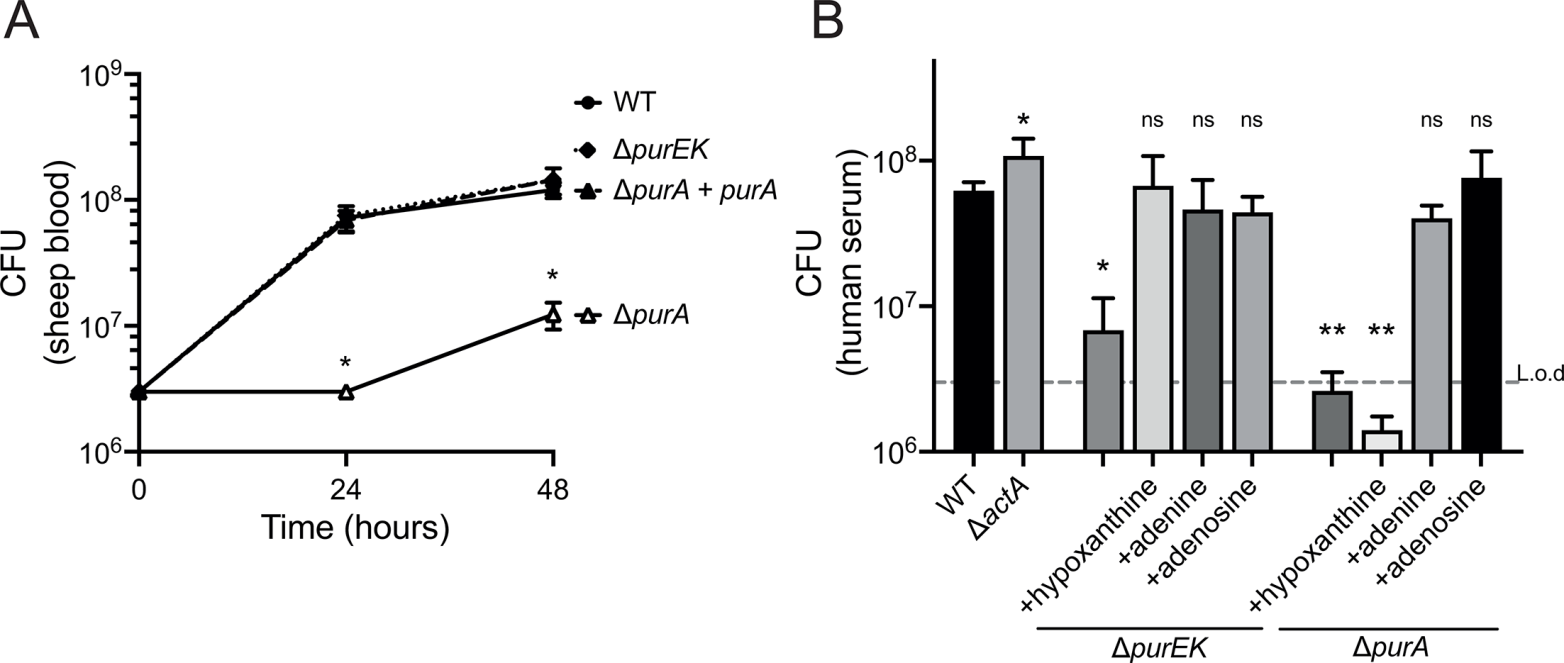
Growth of *L. monocytogenes* purine auxotrophs in blood and serum. Mid-log bacteria were inoculated into 3 mL culture at a density of 1 × 10^6^ CFU/mL. Growth was measured by plating on brain heart infusion agar after incubation in a 37°C shaker. (**A**) Growth in defibrinated sheep blood at 24 and 48 hours. (**B**) Growth in sterile human serum at 24 hours. Dashed line indicates the limit of detection (L.o.d) relative to the starting CFUs. Hypoxanthine, adenine, and adenosine were added into serum at a concentration of 1 mM. Three independent experiments were combined. One-way analysis of variance with multiple comparisons to WT was performed. ns, not significant. *P < 0.05, **P < 0.01.

*L. monocytogenes* is a facultative intracellular pathogen that can grow extracellularly during systemic infections (7, 30). Certain nutrients, including nucleotides, are scarce in blood and need to be synthesized *de novo* by growing bacteria (13). We hypothesized that *de novo* purine biosynthesis is necessary for *L. monocytogenes* growth in blood. Accordingly, we cultured the Δ*purEK* and Δ*purA* mutants in defibrinated sheep blood. After 24 hours of growth, the amount of the WT and Δ*purEK* strains increased by more than 20-fold, while the Δ*purA* mutants displayed negligible growth (Fig. 3A). When cultured in human serum, both purine auxotrophs, Δ*purEK* and Δ*purA*, showed significant growth defects compared to the WT strain (Fig. 3B). The growth defect of Δ*purEK* was fully rescued by exogenous hypoxanthine or adenine/adenosine, while the Δ*purA* mutants were only rescued by adenine/adenosine, similar to Δ*purA* growth in synthetic media (Fig. 3B and Fig. S1). Unlike Δ*purA* mutants, which did not grow in human serum, the Δ*purEK* mutants showed detectable growth, suggesting that there may be a limited amount of hypoxanthine in human serum to support Δ*purEK* growth.

We also determined the growth of a well-characterized attenuated *L. monocytogenes* strain, Δ*actA*, that harbors the primary attenuating mutation exploited in most *L. monocytogenes*-based vaccine strains (31). The Δ*actA* mutants grew similarly to WT in human serum (Fig. 3B), illustrating the safety risk posed by the extracellular growth of *L. monocytogenes* (5, 8).

### Δ*purA* shows distinct growth and localization patterns *in vivo* compared to Δ*actA*

**Fig 4:**
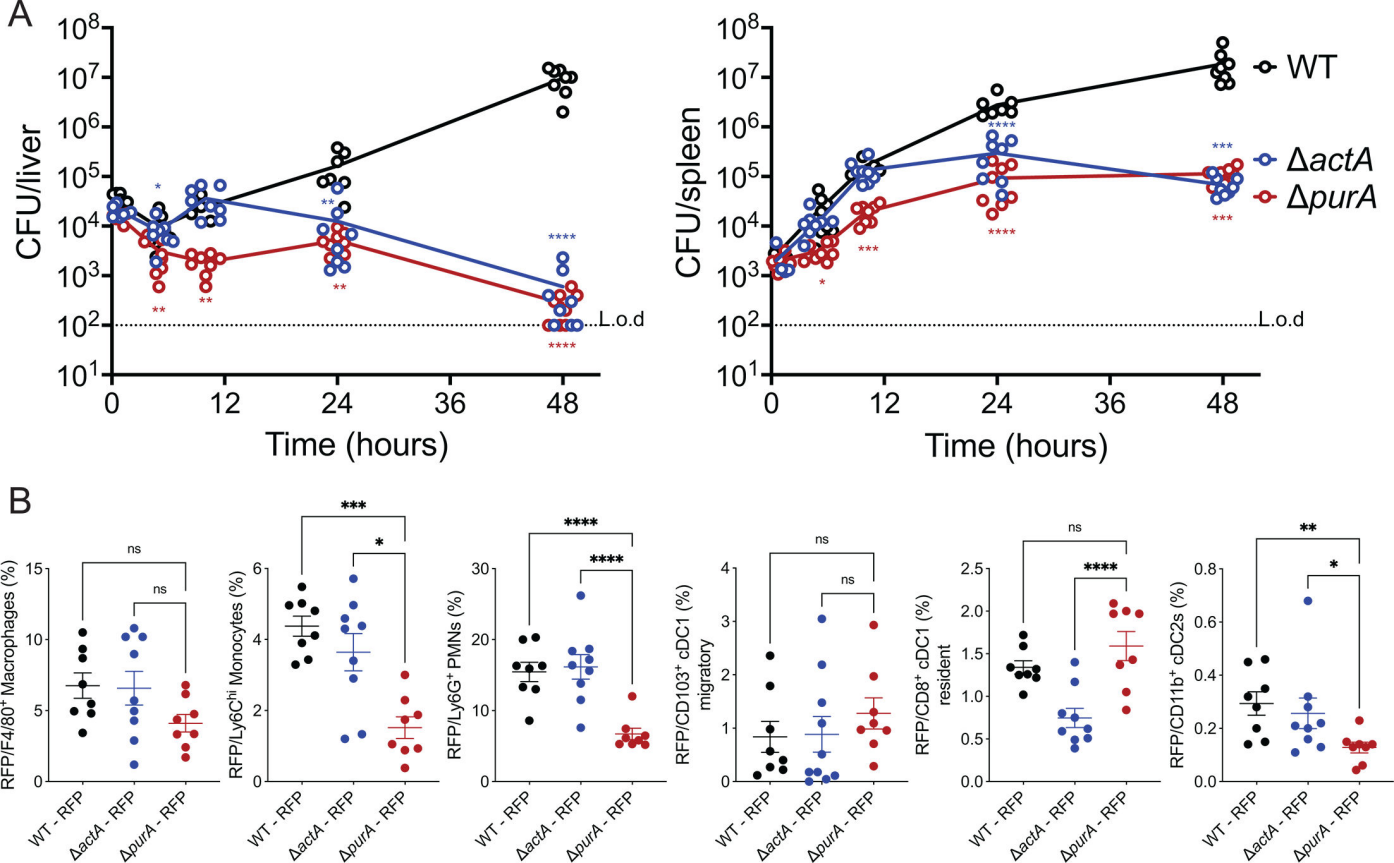
Δ*purA* mutants are attenuated in mice and preferentially localize to specific immune cells in the spleen. (**A**) The *in vivo* growth curve in mice. Eight-week-old CD-1 mice were infected i.v. with 1 × 10^5^ CFU of indicated strains. Bacterial burdens in livers (left) and spleens (right) were measured at 0.5, 5.0, 10.0, 24.0, and 48.0 hour post-infection. The dashed lines indicate the limit of detection (L.o.d.) in each organ. Two biological repeats were combined with a total of seven to eight mice per strain. One-way analysis of variance with multiple comparisons to WT was performed. (**B**) C57BL/6 mice were infected i.v. with 1 × 10^8^ of RFP-expressing *L. monocytogenes* strains. Spleens were harvested 4 hours post-infection and analyzed by flow cytometry. The frequency of splenic immune cells that colocalized with RFP^+^ *L. monocytogenes* strains is shown. A detailed grating strategy is described in Fig. S4. Results represent two biological repeats with three to five mice/group per experiment. Mean ± SEM is shown, and statistical significance was calculated using Student's *t*-test. *P < 0.05, **P < 0.01, ***P < 0.001, ****P < 0.0001.

Given that the Δ*purA* mutants did not grow in BMMs or blood *in vitro*, we hypothesized that the mutants would be highly attenuated *in vivo*. To test this hypothesis, we monitored the growth of the Δ*purA* strain subsequent to intravenous (i.v.) infection during a time course in mice. For comparison, we included a Δ*actA* strain that is approximately 1,000-fold less virulent than the WT but retains immunogenicity (31). We found that while the Δ*actA* mutants grew like WT in both the livers and spleens during the first 12 hours of infection, the Δ*purA* strain failed to grow in the liver and showed some growth in the spleen, but less than WT or Δ*actA* mutants. Between 12 and 24 hours, colony-forming units (CFUs) of the Δ*actA* strain dropped in the liver, whereas CFUs of the Δ*purA* strain increased in both livers and spleens. After 24 hours, while WT bacteria continued to grow, both the Δ*actA* and Δ*purA* mutants decreased in CFUs, and by 48 hours, both mutants had the same number of CFUs (Fig. 4A; Fig. S3). We hypothesized that the difference in growth kinetics that distinguished Δ*purA* from Δ*actA* early during the infection was mediated by the distinct localization and/or growth of these mutants in immune cells. To test this hypothesis, we determined the immune cell tropism of the mutant strains in the spleen at 4 hours post-i.v. infection (Fig. 4B). We focused our analysis on *L. monocytogenes* infection in the spleen because Δ*purA* mutants grew mostly in spleens during the first 24 hours after infection (Fig. 4A), and the spleen is known to play an important role in generating early immune response to *L. monocytogenes*. Furthermore, we used a higher inoculation dose (1 × 10^8^) to reach a sufficient number of CFUs in the spleen at 4 hours post-infection. We constructed *L. monocytogenes* strains expressing RFP under the control of the *actA* promoter (P*_actA_*-RFP), which drives RFP expression specifically upon bacterial entry into the host cell cytosol (25). The compartmentalization of RFP^+^ *L. monocytogenes* in different immune cells in the spleen was determined by flow cytometry using a panel of well-established surface markers for monocytes (Ly6C^hi^), macrophages (F4/80^+^), polymorphonuclear leukocytes (PMNs) also known as neutrophils (Ly6G^+^), and dendritic cells (cDCs), including migratory (CD103^+^) cDC1, resident (CD8^+^) cDC1, and cDC2 (CD11b^+^) (32, 33). During the early stage of infection, all three strains were found in F4/80^+^ macrophages, suggesting that macrophage growth conditions *in vivo* may differ from the ones tested *in vitro* (Fig. 4B and 2A). However, both WT and Δ*actA* RFP^+^ *L. monocytogenes* were more enriched in Ly6C^hi^ monocytes and Ly6G^+^ PMNs compared to Δ*purA* (Fig. 4B). Interestingly, the Δ*purA* mutants showed a unique colocalization pattern within different cDC subsets. Migratory CD103^+^ cDC1 captured each of the three RFP-expressing *L. monocytogenes* strains equally, whereas the frequency of Δ*purA* in CD11b^+^ cDC2s was lower compared to WT and Δ*actA*. Most notably, the Δ*purA* strain colocalized with a significantly higher frequency within resident CD8^+^ cDC1 compared to Δ*actA*. Together, these results suggested that the tropism of the Δ*purA* strain in the spleen is distinct from WT or Δ*actA*, which may influence the induction of immunity.

### The Δ*purA* mutants elicited a CD8^+^ T-cell response and protective immunity

**Fig 5:**
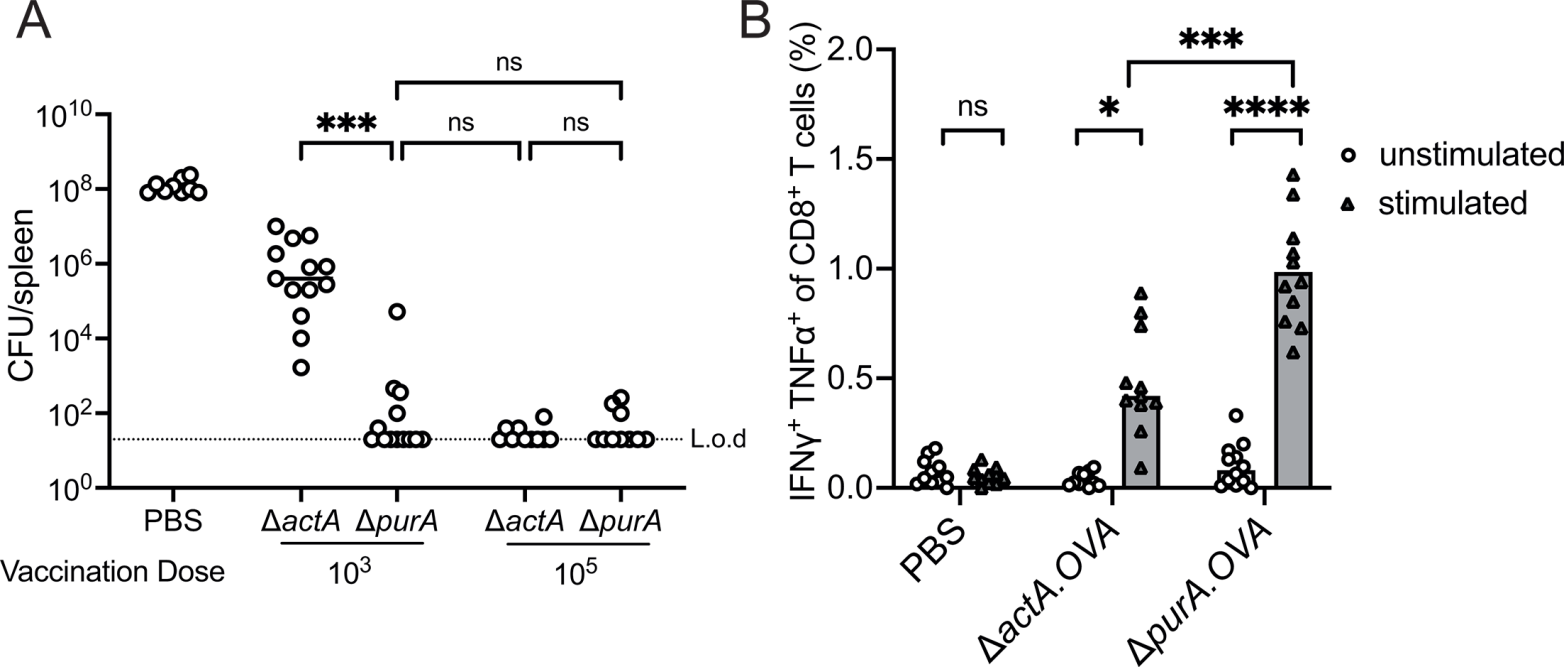
*L. monocytogenes* mutants lacking *purA* stimulate potent cell-mediated immunity. (**A**) Vaccination with *L. monocytogenes* Δ*purA* and Δ*actA* mutants. C57BL/6J mice were vaccinated i.v. with 10^3^ or 10^5^ CFU of *L. monocytogenes* mutants. Four weeks post-vaccination, mice were challenged i.v. with 5 × 10^4^ CFU of WT *L. monocytogenes*. Three days post-challenge, WT CFUs were enumerated from the spleens. Data are pooled from three independent experiments for the 10^3^ group and two experiments for the 10^5^ group. Lines represent medians, and each circle represents individual mouse. (**B**) C57BL/6J mice were infected i.v. with 10^3^ CFU of indicated OVA-expressing strains. Seven days post-infection, single-cell suspensions of splenocytes were stimulated with an OVA-specific peptide followed by intracellular cytokine staining. Data are pooled from three independent experiments. For A and B, one-way analysis of variance with multiple comparisons was performed. L.o.d., limit of detection; ns, not significant; OVA, ovalbumin. *P < 0.05, ***P < 0.001, ****P < 0.0001.

Considering that Δ*purA* had altered growth kinetics and was associated with different splenic cellular niches when compared to Δ*actA*, we wondered if the growth of Δ*purA* in the spleens can lead to CD8^+^ T-cell response and provide protective immunity. Accordingly, C57BL/6J mice were immunized with either 10^3^ or 10^5^ CFU of the Δ*purA* mutants by i.v. injection. Phosphate-buffered saline (PBS) was used as a negative control, and Δ*actA* mutants served as a vaccinated control, as previous reports demonstrated that 10^5^ CFU of the Δ*actA* strain immunized better compared to a lower dose of 10^3^ CFU (34). Four weeks after immunization, mice were challenged with a lethal dose of WT *L. monocytogenes*. Our results showed that 10^5^ CFU of Δ*purA* mutants induced full protection against WT *L. monocytogenes* phenocopying Δ*actA* immunization data reported previously (Fig. 5A) (35). Strikingly, even at a low dose, the Δ*purA* strain conferred strong protection, indistinguishable from the high-dose group (Fig. 5A). Although the Δ*actA* and Δ*purA* mutants were similarly attenuated during infection (Fig. S3), the Δ*purA* mutant was a more potent vaccine at a lower immunization dose (Fig. 5A).

To further evaluate the immunogenicity of the Δ*purA* mutants, we determined the induction of effector CD8^+^ T cells in response to the low-dose bacterial challenges. We introduced the pPL2 integration vector expressing ovalbumin (OVA) fused to the promoter and N-terminus of listeriolysin O (31, 35, 36) in the Δ*purA* and Δ*actA* mutants, and the percentage of OVA-specific interferon‐gamma (IFN‐γ) and tumor necrosis factor alpha (TNF-α) expressing CD8^+^ T cells was determined by flow cytometry. Both the Δ*purA* and Δ*actA* mutants elicited significant antigen-specific effector CD8^+^ T cells upon a single vaccination, but consistent with the findings in Fig. 5A, the Δ*purA* mutants induced a more robust CD8^+^ T-cell response compared to Δ*actA* mutants (Fig. 5B).

## DISCUSSION

The results of this study show that deletion of *L. monocytogenes purA* affects bacterial survival and growth *in vitro* and *in vivo*. Despite being severely attenuated *in vivo*, Δ*purA* mutants elicit a protective CD8^+^ T-cell response. The unique properties of Δ*purA* that combine lack of extracellular growth and enhanced immunogenicity make it a promising candidate therapeutic strain for use in clinical trials for cancer immunotherapies.

Efforts to design *L. monocytogenes*-based cancer vaccine platforms have focused on attenuating *L. monocytogenes* strains to ensure safety in clinical application (5). One of the best-characterized attenuation strategies includes limiting bacterial spread using *actA* mutants as demonstrated in *Listeria-*attenuated double-deleted-based vaccine trials (31, 34, 37). ActA mutants escape from phagosomes like WT but are unable to spread cell-to-cell and have an approximately 3-log defect in mice infections. The major safety challenge posed by the attenuated strains is extracellular growth of *L. monocytogenes* that was detected on ports, implants, and in blood in a few cancer trials (7). Unlike Δ*actA*, Δ*purA* displays limited growth in human serum, indicating that Δ*purA* may offer a superior vaccine safety profile by minimizing bacterial burdens and facilitating *L. monocytogenes* clearance post-immunization.

Comparison between Δ*purA* and Δ*actA* showed that both strains had a comparable virulence defect *in vivo* with minimal growth primarily in the spleens during the first 48 hours of infection (Fig. 4A; Fig. S3). Despite similar attenuation profiles, Δ*purA* led to a more potent CD8^+^ T-cell immune response subsequent to immunization (Fig. 5), suggesting that *purA* may employ alternative ways to activate T cells. Immune interactions early during the infection may result in differential activation of antigen-presenting cells and variable induction of CD8^+^ T-cell response (38, 39). In line with this observation, the distribution of Δ*purA* and Δ*actA* within the splenic environment revealed the preferential localization of the mutants in different immune cell types (Fig. 4B). Macrophages emerged as a common niche for WT, Δ*actA*, and Δ*purA*. Although we detect Δ*purA* mutants in the macrophage cytosol, our experiment cannot conclude whether *in vivo* Δ*purA* mutants are replicating in the cytosol or exhibit limited cytosolic growth as observed in BMMs *in vitro* (Fig. 2A).

The absence of extracellular growth may also contribute to shaping a unique Δ*purA*-driven potent protective immunity. The presence of extracellular *L. monocytogenes* leads to a local inflammatory response and recruitment of PMNs and inflammatory monocytes (40, 41). Therefore, it is possible that the absence of Δ*purA* growth in the extracellular environment dampens the inflammatory response and affects the recruitment of inflammatory leukocytes. Consistent with this hypothesis, Δ*purA* colocalized with inflammatory monocytes and neutrophils to a lesser degree than Δ*actA* (Fig. 4B). Of note, it was surprising to see the high percentage of WT and Δ*actA L. monocytogenes* localized to PMNs, which have long been considered essential for phagocytosis and clearance of bacteria early during infection (42). However, *L. monocytogenes* has been observed to localize to PMNs in mesenteric lymph nodes and gut lamina propria in a foodborne model of listeriosis (43, 44). *L. monocytogenes* has also been shown to reside in the phagosomes of PMNs in a viable but non-culturable state leading to persistent *L. monocytogenes* that is able to spread and grow in other cell types (45). The P*_actA_*-RFP system used in our analysis is designed to detect cytosolic bacteria where P*_actA_*-dependent expression increases approximately 100-fold (46, 47). However, we cannot exclude the possibility that in addition to cytosolic bacteria in PMNs (Fig. 4B), there could also be a phagosomal presence of non-growing *L. monocytogenes*.

Unlike Δ*actA*, Δ*purA* mutants preferentially localized to resident cDC1 similar to WT. CD8^+^ DCs tropism of Δ*purA* raises an interesting possibility that these DCs may be more effective at T-cell priming and expansion contributing to Δ*purA* potent immunogenicity. Indeed, CD8^+^ cDC1 has been shown to be important in *L. monocytogenes* replication and spread in the spleen (48–50). Furthermore, CD8^+^ cDC1 has a delayed phagosomal acidification mechanism, which increases their antigen cross-presentation capacity compared to other DCs (51). The efficient cross-presentation by CD8^+^ DC1s may also explain why Δ*purA* is effective at generating potent cell-based immunity even at lower doses (Fig. 5). A Δ*purA* vaccine candidate may minimize the number of bacteria needed to be administered during vaccination while still maintaining a sufficient amount of antigen during the formation of the immune response.

Our study provides a comprehensive characterization of Δ*purA* growth *in vitro* and *in vivo*, but the precise molecular mechanisms that render Δ*purA* more immunogenic compared to Δ*actA* remain to be determined. One hypothesis is that the presence of ActA contributes to Δ*purA*-dependent activation of immune response. ActA has been shown to be a potent adjuvant in tumor immunotherapy (52). Moreover, infection of resident splenic CD8^+^ DCs was dependent on ActA-mediated cell-to-cell spread (50), consistent with our observation that Δ*purA* and WT localized to CD8^+^ DCs more efficiently than Δ*actA* (Fig. 4B). ActA expression allows *L. monocytogenes* to mediate actin-based motility and protects bacteria from recognition by the autophagy machinery in the host cytosol (29, 53). ActA-dependent escape from autophagic recognition may promote Δ*purA* survival in the splenic niches, resulting in protective immunity. However, our preliminary data show that *actA* alone does not solely account for potent Δ*purA* immunogenicity, as disruption of both *actA* and *purA* (Δ*actA*/*purA::Tn)* does not phenocopy Δ*actA* in an immunization study (Fig. S5). There might be additional aspects of Δ*purA* pathogenesis that enhance its immunogenic properties. For instance, in other species, deletion of *purA* leads to lower levels of c-di-AMP, a major bacterial signaling molecule that is derived from purines (54). Interestingly, high c-di-AMP levels have been shown to downregulate T-cell immunity during *L. monocytogenes* infection (35). Therefore, it is possible that Δ*purA* is defective in c-di-AMP production, which in turn may positively contribute to the development of potent adaptive immunity.

Purines are known to be important in physiology and pathogenesis of many bacterial species including *Staphylococcus aureus*, *Bacillus anthracis*, and *Mycobacterium tuberculosis* (16, 55–57). However, Δ*purA* immunopotency may be unique to *L. monocytogenes* as *purA*-minus mutants in other pathogens, such as *Salmonella* and *Francisella novicida*, failed to induce protective immunity in mice (58–61). Future work will examine Δ*purA* in mouse tumor models. The tumor microenvironment is characterized by high adenosine levels (62, 63), which may enhance the growth of Δ*purA* in the tumor microenvironment even extracellularly. The selective growth of Δ*purA* in tumors could result in a higher bacterial burden and antigen cross-presentation on tumor cells, leading to more efficient tumor-targeting T-cell immunity. It would be beneficial to further examine the safety, kinetics, and cell tropism of Δ*purA* in the tumor microenvironment using i.v. and intratumoral cancer vaccination models (64). Characterizing the efficacy of the attenuated Δ*purA* vaccine candidate in preclinical cancer models will provide valuable insight into the immunogenic capacity of *Listeria*-based immunotherapies.

## MATERIALS AND METHODS

### Bacterial growth conditions

All *L. monocytogenes* strains used in this study were derived from WT strain 10403S (Table 1). Strains were grown in filter-sterilized brain heart infusion (BHI) medium (BD Biosciences). When needed, bacterial culture media supplements were used at the following concentrations: streptomycin at 200 µg/mL, chloramphenicol at 7.5 µg/mL, carbenicillin at 100 µg/mL, and tetracycline at 2 µg/mL. The *Listeria* synthetic medium (LSM) was made using a previously described recipe without adenine (24). When needed, adenine, adenosine, and hypoxanthine were added to the medium at desired concentrations. All reagents were purchased from Sigma unless specified.

**TABLE 1:** List of strains

Strain number	Background	Strain name	Plasmid	Reference
	*Escherichia coli* SM10	NA^*a*^	NA	(65)
DP-E7550	*E. coli* SM10	NA	pKSV7x.Δ*purA*	This study
DP-E7554	*E. coli* SM10	NA	pPL2.LLO441-OVA	This study
	*L. monocytogenes* 10403S	NA	NA	(66)
DP-L7551	*L. monocytogenes* 10403S	Δ*purA*	NA	This study
DP-L7552	*L. monocytogenes* 10403S	Δ*purA + purA*	NA	This study
DP-L1942	*L. monocytogenes* 10403S	Δ*actA*	NA	(67)
DP-L6508	*L. monocytogenes* 10403S	WT-RFP	NA	(68)
DP-L7706	*L. monocytogenes* 10403S	Δ*purA*-RFP	NA	This study
	*L. monocytogenes* 10403S	Δ*actA*-RFP	NA	(31)
DP-L7555	*L. monocytogenes* 10403S	Δ*purA*.*OVA*	NA	This study
DP-L5217	*L. monocytogenes* 10403S	Δ*actA*.*OVA*	NA	(69)
DP-L7703	*L. monocytogenes* 10403S	Δ*actA/purA::Tn*	NA	This study

^*a*^ NA,not applicable.

LSM broth growth curves were obtained with *L. monocytogenes* strains from overnight cultures grown at 37°C with agitation (220 rpm). Overnight bacteria cultures were diluted to an optical density at 600 nm (OD_600_) of 0.05 in a fresh media, and the growth was monitored spectrophotometrically.

### Plasmid and strain construction

Plasmids were introduced into *L. monocytogenes* by conjugation using a donor *E. coli* SM10 and a compatible *L. monocytogenes* strain. In-frame deletion of the *purA* gene was performed using allelic exchange as previously described (70). The complemented strains were generated by integrating a pPL2x vector encoding PurA under the control of the *purA* promoter into mutant strain genomes and selecting for tetracycline-resistant trans-conjugants (71). The OVA strain was generated by the integrative pPL2 vector encoding OVA fused with the promoter and N-terminal of listeriolysin O as described before (32, 34, 37). Δ*purA*-RFP was generated by integrating pPL2 vector encoding P*_actA_*-RFP into the Δ*purA* strain (68). Trans-conjugants were selected for chloramphenicol resistance.

### Intracellular growth curves in BMMs

BMMs were prepared by collecting bone marrow from 8-week-old female C57BL/6J mice (The Jackson Laboratory) and differentiated as previously described (24). All BMMs used in the experiments were cultured in Dulbecco’s modified Eagle’s medium (DMEM) with 20% fetal bovine serum (FBS), 10% macrophage colony-stimulating factor, 1% L-glutamine, 1% sodium pyruvate, and 14 mM 2-mercaptoethanol (Thermo Fisher Scientific). One day prior to infection, 3 × 10^6^ BMMs were plated in 60 mm non-tissue culture-treated dishes (MIDSCI) containing 14 12 mm glass coverslips (Thermo Fisher Scientific). *L. monocytogenes* strains were grown overnight at 30°C without agitation. On the day of infection, bacteria were diluted in sterile PBS, and a multiplicity of infection (MOI) of 0.25 was used for infection. At 30 min post-infection, BMMs were washed twice with PBS. At 1 hour post-infection, 50 µg/mL gentamicin was added to the cell culture media to prevent bacteria from growing extracellularly. Bacteria growth was enumerated by plating CFUs from three coverslips at each desired time point.

### Phagosome escape and immunofluorescence analysis

The p62 colocalization-dependent escape assay and immunofluorescence analysis were performed as described before (28). In brief, 2 × 10^5^ BMMs were seeded on a sterile 12 mm coverslip in a well of a 24-well plate containing 500 µL of BMM medium and cultured overnight. BMMs were incubated with 250 ng/mL of cytochalasin D 30 min before infection to prevent actin polymerization. *L. monocytogenes* strains were grown in BHI broth at 37°C with agitation until an OD_600_ of 0.5–1.0 was obtained. Cells were infected with exponential phase bacteria at an MOI of 5–10 for 30 min and fixed in 4% paraformaldehyde at 1.5 hour post-infection. Permeabilized cells were immunostained using antibodies recognizing *L. monocytogenes* (1:1,000 dilution, BD Biosciences) and p62 (1:200 dilution; Fitzgerald 20R-PP001), and then secondary antibodies were conjugated with fluorophores (1:2,000 dilution, Invitrogen). Coverslips were mounted in ProLong Gold antifade reagent with 4′,6-diamidino-2-phenylindole (Invitrogen) and imaged with a BZ-X710 KEYENCE fluorescent microscope using a ×100 objective. More than 100 bacteria were measured for each tested condition. Images were analyzed using ImageJ (National Institutes of Health).

### Plaque assay

Plaque assays were performed as previously described (72). In brief, L2 fibroblasts were propagated in high-glucose Gibco DMEM (Thermo Fisher Scientific) plus 10% FBS (Avantor-Seradigm), 1 mM sodium pyruvate (Corning), and 2 mM L-glutamine (Corning) were plated 1.2 × 10^6^ cells/well in a six-well tissue-culture-treated plate and incubated overnight. *L. monocytogenes* strains were grown at 30°C overnight without agitation. Bacteria were diluted 1:10,000 in prewarmed media and overlaid on host cells. After 1 hour, the cells were washed with PBS twice and overlayed with media plus 0.7% agarose and gentamicin at 10 µg/mL. Cells were stained with Neutral Red for at least 6 hours prior to imaging 3 days post-infection. Plaque areas were measured using ImageJ software (73), collecting more than four plaques per strain per experiment.

### Growth in sheep blood and human serum

Growth in sheep blood was performed as described (10). Fresh defibrinated sheep blood was purchased from HemoStat Laboratories. *L. monocytogenes* strains were grown in BHI broth at 37°C with agitation until an OD_600_ of 0.5–1.0 was obtained. Bacteria were washed once with sterile PBS and resuspended in 3 mL to a density of 1 × 10^6^ CFU/mL in prewarmed sheep blood or sterile human serum (Sigma, H4522), which was heat-inactivated and buffered at pH 7 with 5 mM HEPES. The blood cultures were incubated at 37°C with agitation (220 rpm) for 24 hours, and CFUs were plated on BHI agar by serial dilutions. Hypoxanthine, adenine, and adenosine were added to a final concentration of 1 mM.

### Mouse infections

Eight-week-old CD-1 mice (Charles River) were infected intravenously with 1 × 10^5^ CFU of desired *L. monocytogenes* strains in 200 µL of PBS. Animals were sacrificed at different time points during a 48 hour infection, and spleens and livers were harvested in 5 or 10 mL 0.1% IGEPAL CA-630 in water, respectively, and plated for enumeration of bacterial burdens.

### Flow cytometry analysis of cells infected with *L. monocytogenes in vivo*

Eight-week-old C57BL/6 mice (Jackson Laboratories) were infected intravenously with 1 × 10^8^ CFU of *L. monocytogenes* strains in 200 µL of PBS. Four hours post-infection, mice were sacrificed, and spleen tissues were collected. Spleens were digested in 5 mL of digestion mix (RPMI Sigma, Collagenase D 1 mg/mL, DNase I 20 µg/mL, FBS 2%) for 30 min at 37°C. Single-cell suspension was generated by passing the digested mix through a 70 µm nylon filter mesh (FLACON) and washing the filter with 15 mL of PBS. The suspension was centrifuged, and the resulting pellet was resuspended in 1 mL of ammonium-chloride-potassium lysis buffer (150 mM NH_4_Cl, 10 mM KHCO_3_, 0.1 mM Na_2_EDTA, pH 7.3). Following a 3 min incubation, the suspension was centrifuged again and resuspended in 400 µL PBS containing 4 mM EDTA and 2% FBS. Two hundred microliters of the resulting cell suspension was loaded onto a 96-well plate and centrifuged. The pellet was resuspended in 200 µL PBS containing 0.5% FC blocking solution (BioLegend, #101320) and incubated for 20 min at 4°C. The cells were centrifuged and resuspended in 100 µL staining solution containing the antibodies specific to the surface marker on the immune cells. The suspension was incubated for 20 min at 4°C and washed twice with 200 µL of PBS. The pellet collected was resuspended in 4% PFA and incubated for 10 min at room temperature. Cell suspension was washed with 100 µL of PBS twice prior to analysis. Viability staining was performed using Live/Dead Fixable Blue (Molecular Probes). A detailed list of flow cytometry staining markers used for these experiments can be found in Table S1. Forward scatter (FSC) and side scatter (SSC) were used to exclude cell debris, and FSC-A and FSC-H were used to exclude cell aggregates. RFP^+^ *L. monocytogenes* localization to specific immune cell populations was determined using Δ*actA L. monocytogenes* as a negative gating control. Flow cytometry was performed on a BD LSR Fortessa X20 or LSRFortessa (BD Biosciences) or CyTEK Aurora (CyTEK Bioscience). Resulting data sets were analyzed using FlowJo software (Tree Star).

### Vaccination of mice

Eight-week-old female C57BL/6J mice (The Jackson Laboratory) were vaccinated by intravenous injection of *L. monocytogenes* in 200 µL PBS. Four weeks post-vaccination, mice were challenged with 5 × 10^4^ CFU WT *L. monocytogenes* injected intravenously in 200 µL PBS. Three days post-challenge, mice were euthanized, and spleens were harvested, homogenized in 0.1% IGEPAL CA-630 (Sigma), and plated on LB agar with 200 µg/mL streptomycin for enumeration of bacterial burdens.

### Antigen-specific T-cell response and flow cytometry

The frequency of IFN-γ and TNF-α secreting CD8^+^ T cells specific for OVA was determined by intracellular cytokine staining as described before (34). Eight-week-old female C57BL/6J mice (The Jackson Laboratory) were infected by intravenous injection of *L. monocytogenes* in 200 µL PBS. Seven days post-infection, single-cell suspensions of splenocytes were stimulated with OVA 257-264 peptide epitope (SIINFEKL, InvivoGen vac-sin) for 4 hours in the presence of GolgiPlug protein transport inhibitor (BD Biosciences). Cells were then stained for viability and cell-surface markers, CD8 and CD4, and fixed in 2% paraformaldehyde. Intracellular cytokine staining for IFN-γ and TNF-α was performed in permeabilized cells following the user manual of the Fixation/Permeabilization Solution Kit (BD Biosciences). Antibodies were purchased from eBiosciences (Table S1). Samples were analyzed on an LSR Fortessa (BD Biosciences), gated by FSC-A and SSC-A to exclude debris, FSC-H, and FSC-A for single cells. Data analysis was performed using FlowJo (Treestar).

### Statistical analysis

All statistical analyses were performed using GraphPad Prism version 9.2 for Windows (GraphPad Software, La Jolla, CA, USA; https://www.graphpad.com/).

## SUPPLEMENTAL MATERIAL

Supplemental material iai.00343-25-s0001.docx Fig. S1 to S5; Table S1.

ASM does not own the copyrights to Supplemental Material that may be linked to, or accessed through, an article. The authors have granted ASM a non-exclusive, world-wide license to publish the Supplemental Material files. Please contact the corresponding author directly for reuse.
