# Supplementary material for: Listeria monocytogenes adenosine auxotrophs are impaired for intracellular and extracellular growth but retain potent immunogenicity
Source: Infect Immun. 2025 Sep 5;93(10):e00343-25. doi: 10.1128/iai.00343-25 (PMC12519778; doi:10.1128/iai.00343-25)
Supplement: Supplemental material — Fig. S1 to S5; Table S1. [file iai.00343-25-s0001.docx]

**SUPPLEMENTAL MATERIAL**

***Listeria monocytogenes* adenosine auxotrophs are impaired for intracellular and extracellular growth but retain potent immunogenicity.**

**Ying Feng,^a,c*^ Mariya Lobanovska,^a,c^ Jenna Vickery,^a^ Jesse Garcia Castillo,^a**^ Leslie Güereca,^a***^ Shannon K. Chang,^b****^ Michel DuPage,^a^ Daniel A. Portnoy^a,b^#**

**SUPPLEMENTAL FIGURES AND FIGURE LEGENDS: SUPPL FIGURES 1- 5**

**SUPPLEMENTAL TABLE S1**

**SUPPLEMENTAL FIGURE S1**

**Figure S1** The Δ*purA* mutants are auxotrophic for adenine and adenosine

(A) Growth of *L. monocytogenes* strains on LSM agar with or without purines supplementation. Hypoxanthine, adenine and adenosine were added into the media at the concertation of 500 µM.

Growth curve of Δ*purA* mutants in LSM liquid media supplemented with indicated concentration of adenosine (B) and adenine (C). Overnight cultures were normalized to OD_600_=0.05 in fresh media and grown for 24 hours at 37^o^C with agitation.

**SUPPLEMENTAL FIGURE S2**

**Figure S2** Mutants lacking *purA* have no defect in plaque formation

Monolayers of L2 fibroblasts were infected with *L. monocytogenes* for 1 hour. Plaques were measured 3 days post-infection and presented as percentage of WT. Three independent experiments were combined. Data are mean ± SD. Student’s *t*-test; ns, not significant.

**SUPPLEMENTAL FIGURE S3**

**
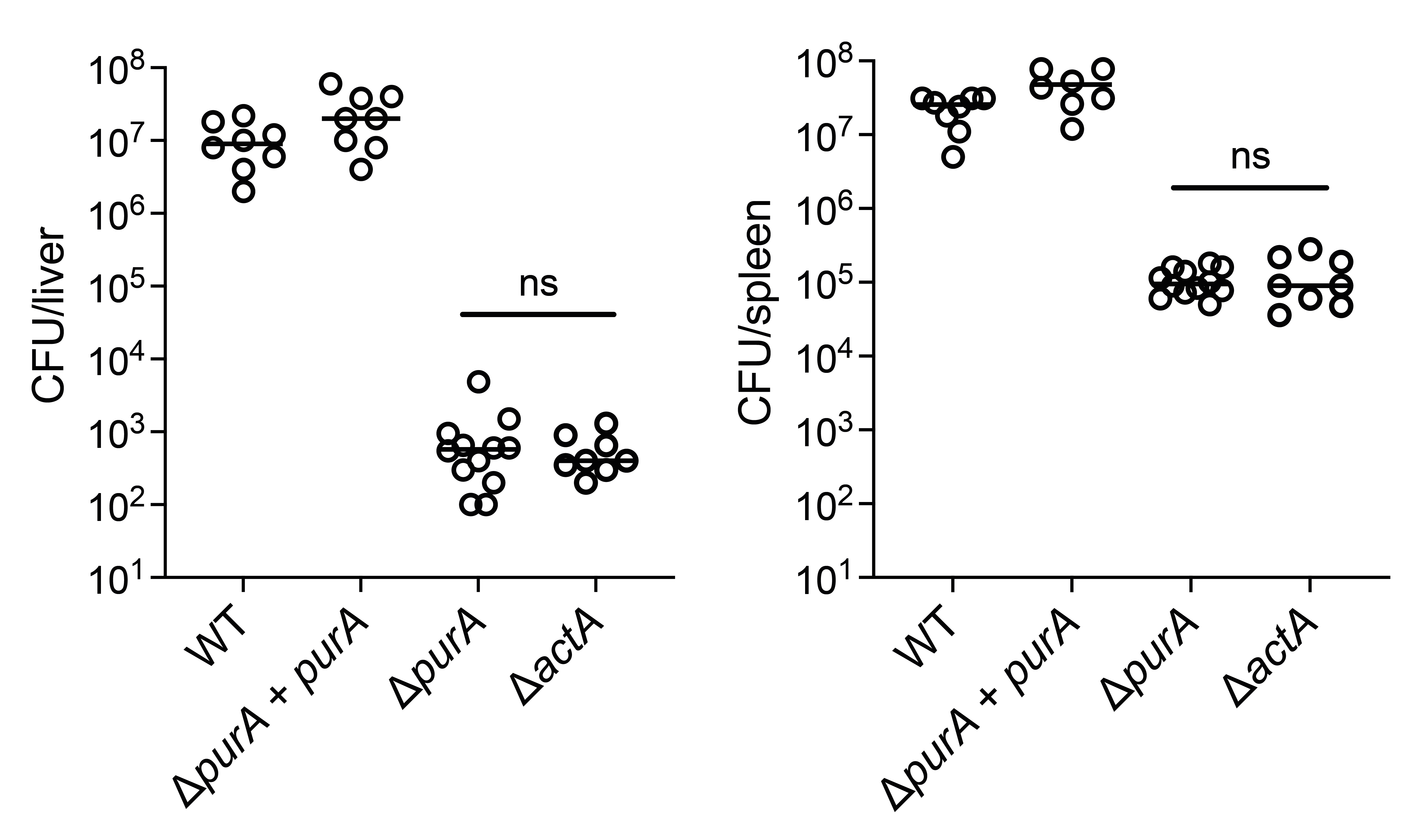
**

**Figure S3** The Δ*purA* mutants are severely attenuated in mice.

Eight-week-old CD-1 mice were infected intravenously with 1 × 10^5^ CFUs of indicated strains. Bacterial burdens in livers and spleens were measured 48 hours post infection. Each circle represents an individual mouse. Lines represent medians. Two biological repeats were combined with a total of 8-10 mice per strain. One-way ANOVA, multiple comparisons; ns, not significant.

**SUPPLEMENTAL FIGURE S4**

**
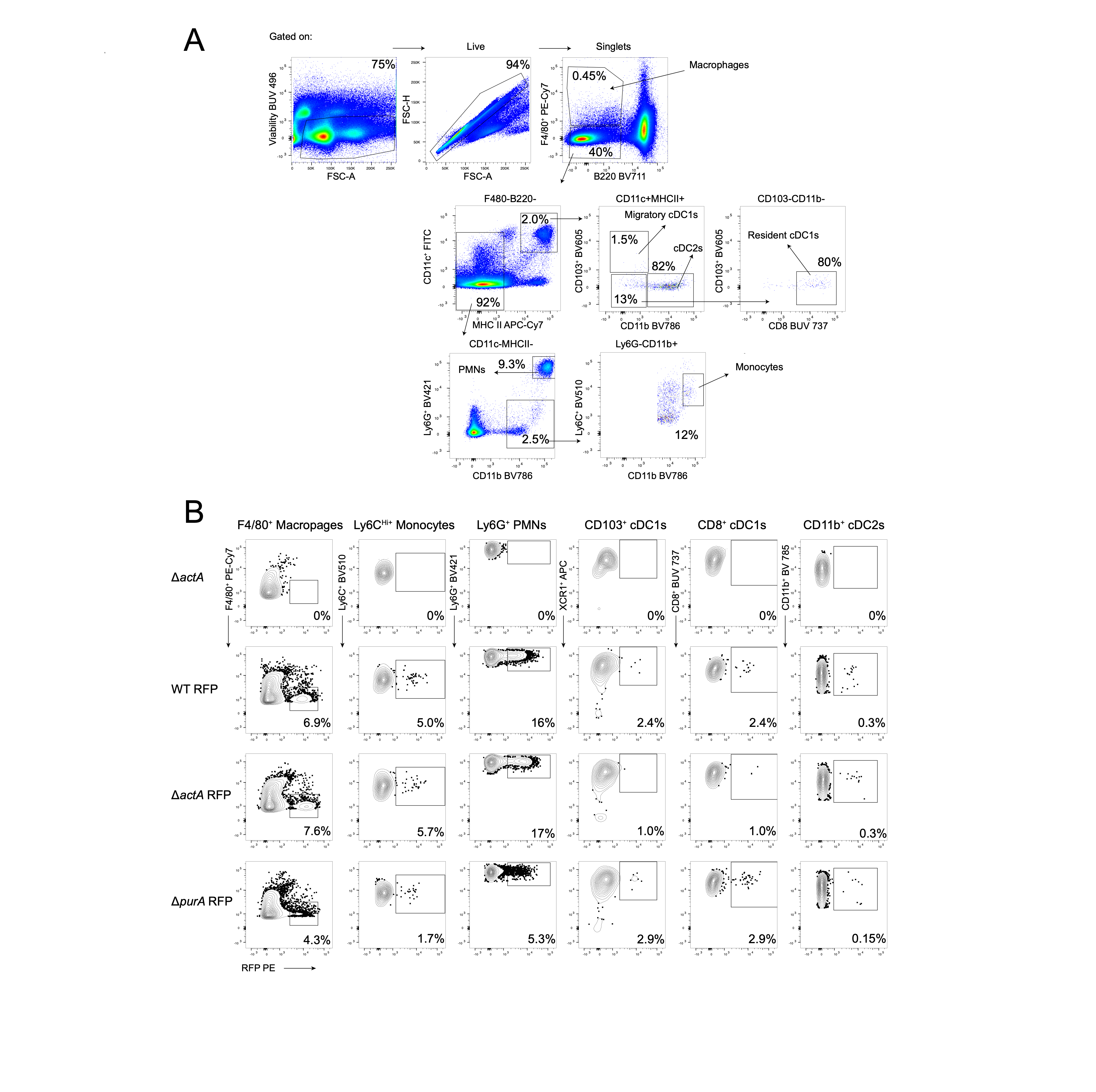
**

**
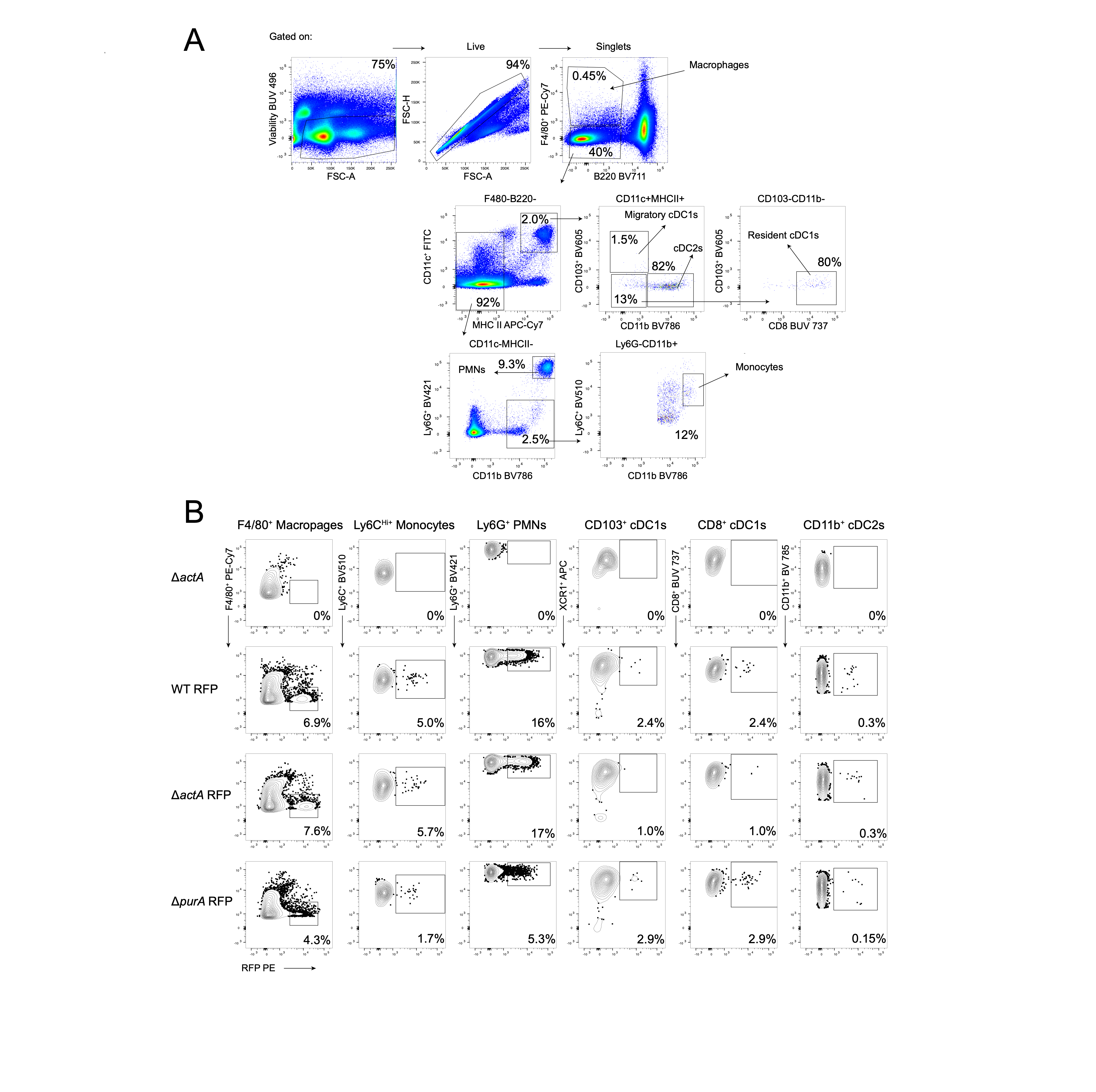
**

**Figure S4** Representative FACS plot gating strategy used for the analysis of flow cytometry data presented in Fig. 4B. A. Gating strategy depicts immune cells in spleens of C57BL/6J mice infected with WT-RFP *L. monocytogenes*. Same strategy was applied for the analysis of splenocytes infected with Δ*actA*-RFP and Δ*purA*-RFP. B. RFP gating strategy used for the analysis of RFP-expressing *L. monocytogenes* localization in splenic immune cells. Δ*actA* was used as a negative control.

**SUPPLEMENTAL FIGURE S5**

**Figure S5** Δ*actA/purA::Tn* immunogenicity *in vivo.*

Eight-week-old C57BL/6J mice were infected intravenously with either 1 × 10^3^ or 1 × 10^5^ CFUs of Δ*actA/purA::Tn* and challenged with 5 × 10^4^ CFUs of WT 28 days post infection. Bacterial burdens in spleens were measured 72-hour post challenge dose administration. Each circle represents an individual mouse. Lines represent medians, L.o.d. is limit of detection. Data represents one biological repeat with 5 mice per strain per group. Δ*actA/purA::Tn* is more immunogenic than Δ*actA* but not as potent as Δ*purA* (Fig. 5A).

**SUPPLEMENTAL TABLE S1**

**Table S1: Flow cytometry cell staining markers**

| Flow cytometry panel used in Figure 4B | | | |
| --- | --- | --- | --- |
| *Marker* | *Channel* | *Cat#* | *Dilution* |
| Viability | BUV496 | Invitrogen #L23105 | 1:500 |
| MHC II | APC-Cy7 | BioLegend #107627 | 1:200 |
| CD11c | FITC | BioLegend #117306 | 1:100 |
| CD11b | BV785 | BioLegend #101243 | 1:200 |
| Ly6C | BV510/AmCyan | BioLegend #128033 | 1:200 |
| B220 | BV711 | BioLegend #103255 | 1:400 |
| F4/80 | PE-Cy7 | BioLegend #123114 | 1:200 |
| Ly6G | e450/PB | BioLegend #127611 | 1:100 |
| RFP | PE |  |  |
| CD103 | BV605 | BioLegend #121433 | 1:100 |
| XCR1 | AF647 | BioLegend #148214 | 1:100 |
| CD8a | BUV737 | Biosciences #612759 | 1:200 |
| SirP-alpha | PerCP-Cy5.5 | BioLegend #144010 | 1:100 |
| Flow cytometry panel used in Figure 5B | | | |
| Viability | APC-Cy7 / BV510 | Invitrogen #L23105 | 1:1000 |
| CD4 | BV605 | BioLegend #100404 | 1:100 |
| CD8 | BUV737 | BioLegend #100704 | 1:100 |
| IFN-γ | FITC | BioLegend #505806 | 1:100 |
| TNF-α | PerCP-Cy5.5 / BB700 | BioLegend #506322 | 1:100 |
